# Predictive value of neutrophil-to-lymphocyte ratio for distant metastasis in gastric cancer patients

**DOI:** 10.1038/s41598-022-14379-4

**Published:** 2022-06-17

**Authors:** Xin Zhang, Xuan Wang, Wenxing Li, Tuanhe Sun, Dongmei Diao, Chengxue Dang

**Affiliations:** Department of Surgical Oncology, First Affiliated Hospital, Medical College, Xi’an Jiaotong University, 277 West Yanta Road, Xi’an, 710061 Shaanxi China

**Keywords:** Cancer, Biomarkers

## Abstract

As a systemic inflammatory marker, the significance of NLR in predicting tumor prognosis and early lymph node metastasis is well known, including gastric cancer (GC). However, whether NLR can reflect GC metastasis status remains to be explored. We retrospectively enrolled 1667 GC patients treated in our hospital from December 2010 to December 2018. Patients were grouped according to the presence or absence of metastases. Receiver operating characteristics (ROC) curve analysis was used to evaluate the diagnostic efficacy of markers in assessing GC metastasis. Then we conducted a joint ROC curve analysis. The effects of clinicopathological parameters on GC metastasis were assessed using multiple logistic regression analysis. 743 (44.6%) patients were diagnosed with metastatic GC. Patients with GC metastases have younger age, higher CEA, CA19-9, CA72-4 and NLR. Based on the comparison of AUC, NLR has diagnostic efficacy comparable to that of GC markers. The AUC of NLR combined with GC markers had significantly higher predicting efficacy than that without combination for assessing peritoneal metastasis (P = 0.013), osseous metastasis (P = 0.017) and hepatic metastasis (P < 0.001). In multiple logistic regression analysis, age, NLR, CEA, CA19-9 and CA72-4 were found to be independently associated with GC metastasis (all P < 0.05). NLR was a risk factor of GC metastasis. Combining CEA, CA19-9, CA72-4 and NLR could better predict metastases in GC.

## Introduction

Gastric cancer (GC) is the second most common cancer in China and the third leading cause of death among all cancer types. About 403,000 new GC cases were reported in 2015, with a total of 291,000 deaths^1^. Because cancer is prone to metastasis and recurrence, the 5-year survival rate of GC patients remains low^2^.

Recent studies have borne out claims that inflammation has a vital role in the development and progression of many diseases, including cancers. Inflammation affects every step of tumorigenesis, from initiation, to tumor promotion, to metastatic progression^3^. Subclinical, often undetectable, inflammation may be equally important in increasing cancer risk (for instance, inflammation caused by diabetes)^4^. As one of the most abundant leukocytes, neutrophils play an essential role in cancer progression^5^. Effects of neutrophils in peripheral blood and tumor microenvironment on GC have been reported^6^. Although tumor-activated neutrophils infiltrating the tumor microenvironment have a significant tumor-promoting effect, they are difficult to obtain. The NLR is a common indicator of systemic inflammation and is defined as absolute neutrophil count divided by absolute lymphocyte count. Cumulative studies have reported that cancer patients have higher NLR levels than healthy people^7^. NLR can act as an independent prognostic factor for many malignant tumors such as lung cancer^8^, colorectal cancer^9^ and GC^10^. NLR is also closely related to tumor metastasis, especially lymph node metastasis (LNM)^11^. Meanwhile, studies have reported that NLR is not an independent risk factor for LNM in patients with early GC gastric cancer^12,13^. NLR is also closely related to distant metastasis of tumor^14^. Although blood cell levels can be routinely obtained, the relationship between blood cell levels and tumor metastasis has not been systematically studied. NLR may be a novel non-invasive and convenient biomarker for detecting tumor metastasis. Therefore, our retrospective study aims to investigate the diagnostic efficacy of NLR for assessing GC metastasis.

## Results

### Patient's characteristics

For the entire cohort, the median age of patients was 61 years (range 54–67) and male accounted for 72.9%. 743 (44.6%) patients were diagnosed with metastatic GC. CEA, CA19-9 and CA72-4 in the metastatic group was significantly higher than that in the non-metastatic group (all P < 0.001). Increased absolute neutrophil count and decreased absolute lymphocyte count in the metastasis group resulted in significantly increased NLR (P < 0.001). In addition, the albumin level of the metastasis group decreased and the globulin level increased (both P < 0.001) (Table 1). Among all patients, 923 patients were diagnosed with primary tumors, 163 with peritoneal metastasis, 119 with osseous metastasis, 38 with pulmonary metastasis, 46 with lymph node metastasis, 192 with hepatic metastasis, 22 with ovarian metastasis, 122 with multisite metastasis, 8 with brain metastasis and 34 with metastasis of other organs.

**Table 1:** Demographic and baseline characteristics of patients.

Characteristic	Overall (*N* = 1667)	Non-metastasis (*N* = 924)	Metastasis (*N* = 743)	P value
**Gender, no. (%)**				< 0.001
Male	1215(72.9)	707(76.5)	508(68.4)	
Female	452(27.1)	217(23.5)	235(31.6)	
Age, year	61(54–67)	61(54–68)	59(52–67)	0.001
CEA, ng/ml	2.82(1.58–7.07)	2.345(1.37–4.19)	3.88(1.94–17.25)	< 0.001
CA199, U/ml	12.46(6.71–36.87)	10.49(5.95–19.96)	18.22(8.31–124.7)	< 0.001
CA724, U/ml	2.98(1.35–9.43)	2.11(1.17–5.56)	5.32(1.85–21.31)	< 0.001
Erythrocyte, 10^12^/l	4.2(3.7–4.6)	4.28(3.8–4.67)	4.07(3.6–4.47)	< 0.001
Hemoglobin, g/l	124(104–140)	129(107–143)	119(102–133)	< 0.001
Leukocyte, 10^9^/l	5.68(4.51–7.11)	5.47(4.4–6.8)	6.04(4.7–7.85)	< 0.001
Neutrophil, 10^9^/l	3.56(2.67–4.82)	3.29(2.48–4.29)	3.97(2.92–5.75)	< 0.001
Lymphocyte, 10^9^/l	1.42(1.08–1.83)	1.53(1.18–1.92)	1.29(0.95–1.67)	< 0.001
NLR	2.45(1.67–3.83)	2.1(1.52–3.02)	3.11(2.08–4.86)	< 0.001
Platelet, 10^9^/l	207(159–263)	205(160–259.75)	209(158–269)	0.317
Monocyte, 10^9^/l	0.4(0.29–0.52)	0.39(0.28–0.5)	0.4(0.3–0.53)	0.018
Albumin, g/l	38.2(34.8–41.4)	38.6(35.4–41.7)	37.7(34–40.9)	< 0.001
Globulin, g/l	26.2(23.4–29.3)	25.3(22.6–28.2)	27.4(24.7–30.5)	< 0.001
**TNM stage**^**a**^				–
I	146(11.6)	146(28.1)	–	
II	56(4.4)	56(10.8)	–	
III	318(25.2)	318(61.2)	–	
IV	743(58.8)	–	743	

Data are shown as number of cases and percentage or median and interquartile range.

*TNM* tumor-node-metastasis, *NLR* neutrophil-to-lymphocyte ratio, *CEA* carcinoembryonic antigen, *CA19-9* carbohydrate antigen 19-9, *CA72-4* carbohydrate antigen 72-4.

^a^404 data missing because some patients were not treated surgically or were treated only with palliative care, resulting in unable to accurately assess the pathological staging.

### NLR can more efficiently predict gastric cancer metastasis than tumor markers

**Figure 1:**
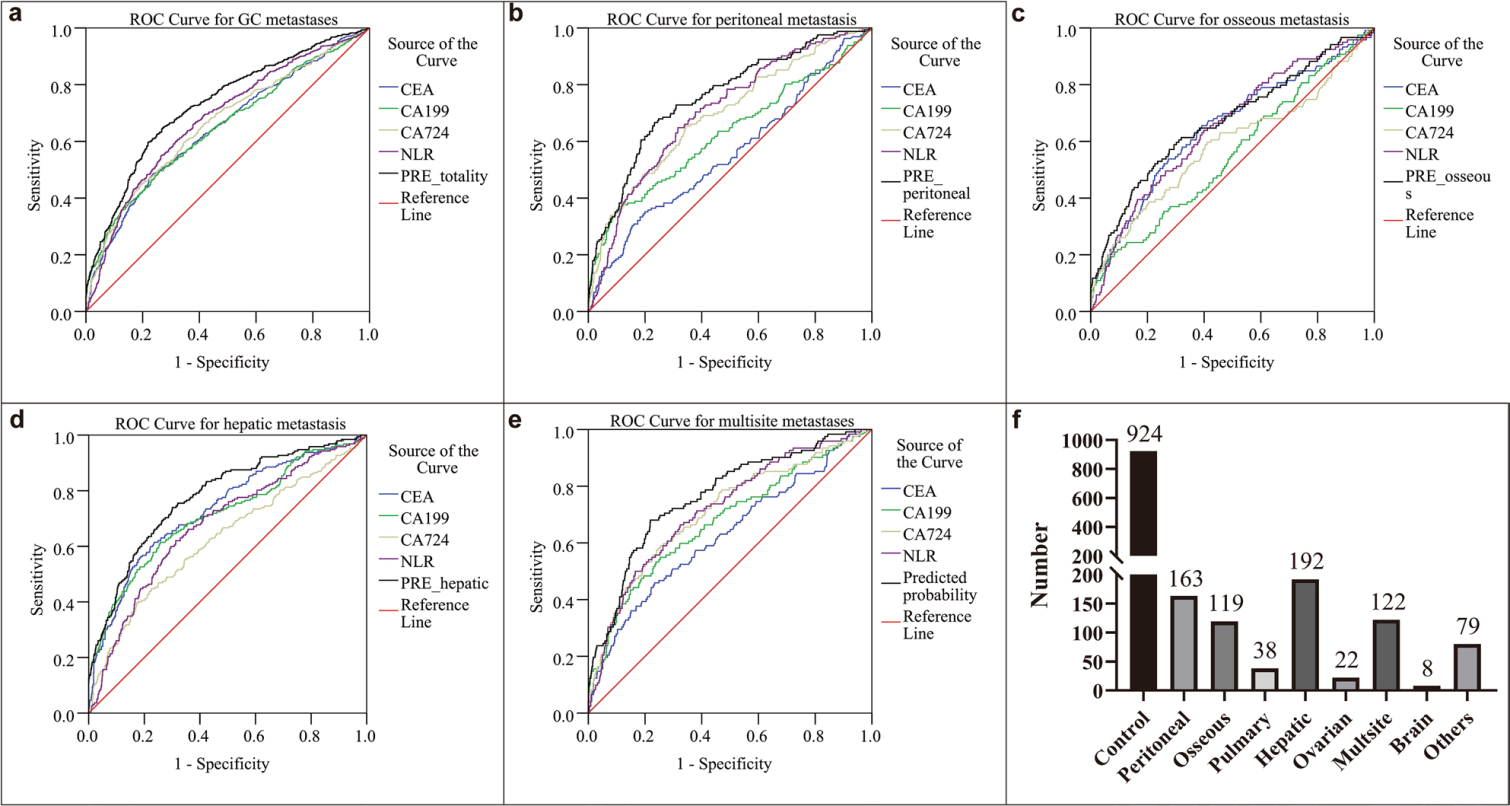
ROC analysis for the prediction of GC metastasis. AUC indicates the diagnostic power of CEA, CA19-9, CA72-4, NLR and prediction probability for total (**a**), peritoneal (**b**), hepatic (**d**), osseous (**c**) and multisite metastasis (**e**). Cases of control group and different sites of metastases (**f**). PRE, prediction probability that was obtained by binary logistic regression of CEA, CA199, CA724 and NLR.

ROC analysis was performed to evaluate the diagnostic efficacy of NLR for predicting GC metastasis. The optimal cutoff values for CEA, CA19-9, CA72-4 and NLR were determined to be 4.34 (with a sensitivity of 48.5% and a specificity of 76.3%), 18.69 (with a sensitivity of 49.7% and a specificity of 74.1), 6.96 (with a sensitivity of 44.7% and a specificity of 80.7%) and 2.91 (with a sensitivity of 55% and a specificity of 73.4%), respectively. The AUC of CEA for predicting GC metastasis was 0.647 [95% confidence interval (CI): 0.62–0.674, P < 0.001], of CA19-9 was 0.649 (95% CI: 0.622–0.675, P < 0.001), of CA72-4 was 0.662 (95% CI: 0.636–0.688, P < 0.001) and of NLR was 0.679 (95% CI: 0.653–0.705, P < 0.001). The predicting efficacy of NLR was comparable to GC markers (Fig. 1a). The predicted probability of combining GC markers (P1) and the predicted probability of combining GC markers and NLR (P2) was obtained by binary logistic regression. The optimal cutoffs of P1 were 0.39 (with a sensitivity of 53.8% and a specificity of 75%) and of P2 was 0.39 (with a sensitivity of 64.9% and a specificity of 72.8%). The AUC of P1 was 0.716 (95% CI: 0.691–0.74, P < 0.001) and of P2 was 0.73 (95% CI: 0.705–0.754, P < 0.001). Although there was no significant difference between the AUC of P1 and P2 (P = 0.209), the combination of NLR slightly increased diagnostic efficacy (Table 2).

**Table 2:** AUC and the cutoff value of diagnostic indicators at the maximum of Youden index for GC metastases (N = 1667).

	AUC	95% CI	Cut-off	Sen	Spe	Youden index	PPV	NPV	P value
NLR	0.679	0.653–0.705	2.91	0.55	0.734	0.284	0.549	0.734	< 0.001
CEA	0.647	0.62–0.674	4.34	0.485	0.763	0.248	0.546	0.719	< 0.001
CA199	0.649	0.622–0.675	18.69	0.497	0.741	0.238	0.537	0.727	< 0.001
CA724	0.662	0.636–0.688	6.96	0.447	0.807	0.254	0.447	0.799	< 0.001
P1	0.716	0.691–0.74	0.39	0.538	0.75	0.333	0.568	0.758	0.209
P2	0.73	0.705–0.754	0.39	0.649	0.728	0.377	0.637	0.733	Ref

*AUC* area under receiver operating characteristics, *CI* confidence interval, *Sen* sensitivity, *Spe* specificity, *PPV* positive predictive value, *NPV* negative predictive value, *P* P value for comparison of AUC of reference with other indicators using DeLong’s test, *P1* prediction probability obtained by binary logistic regression combining CEA, CA199, CA724, *P2* prediction probability obtained by binary logistic regression combining CEA, CA199, CA724 and NLR, *Ref* reference.

We also assessed diagnostic power of NLR for single site metastasis or peritoneal metastasis. ROC analysis was performed based on data stratified by metastasis sites. For peritoneal metastasis (n = 163), AUC (95% CI) of NLR was 0.704 (0.662–0.747), of CEA was 0.557 (0.507–0.608), of CA19-9 was 0.626 (0.573–0.679) and of CA72-4 was 0.694 (0.648–0.739), respectively (Supplementary Table 1). Diagnostic efficacy of prediction probability obtained by binary logistic regression of CEA, CA199, CA724 and NLR was significantly higher than NLR (P = 0.003), CEA (P < 0.001), CA19-9 (P < 0.001) and CA72-4 (P = 0.013) (Fig. 1b). For osseous metastasis (n = 119), AUC (95% CI) of NLR was 0.653 (0.599–0.707), of CEA was 0.65 (0.594–0.707), of CA19-9 was 0.555 (0.498–0.612) and of CA72-4 was 0.586 (0.524–0.648), respectively (Supplementary Table 2). Similar to peritoneal metastasis, diagnostic efficacy of prediction probability was significantly higher than CA19-9 (P < 0.001) and CA72-4 (P = 0.017) except for NLR (P = 0.313) and CEA (P = 0.514) (Fig. 1c). As for hepatic metastasis (n = 192), AUC (95% CI) of NLR was 0.676 (0.633–0.718), of CEA was 0.733 (0.693–0.774), of CA19-9 was 0.713 (0.669–0.757) and of CA72-4 was 0.626 (0.58–0.673) (Supplementary Table 3). Diagnostic efficacy of prediction probability was significantly higher than NLR (P < 0.001), CEA (P = 0.041), CA19-9 (P = 0.002) and CA72-4 (P < 0.001) (Fig. 1d). For multisite metastases, the results of the analysis were similar to that for the overall cohort (Supplementary Table 4, Fig. 1e). We did not analyze other site such as pulmonary metastasis (n = 38) and ovarian metastasis (n = 22) etc. on account of small sample size (Fig. 1f).

Correlation of NLR and other parameters was showed in the Table 3. In GC patients, NLR showed a positive correlation with CEA, CA19-9, CA72-4 (r = 0.157, 0.173 and 0.161, respectively, all P < 0.001) and no correlation of NLR with age (P = 0.205) was found.

**Table 3:** Correlation between NLR and clinicopathological characteristics.

	Spearman correlation	P value
Age	0.031	0.205
CEA	0.157	< 0.001
CA19-9	0.173	< 0.001
CA72-4	0.161	< 0.001

Parameters are grouped by their cutoff values. Multivariate logistic regression analysis showed that younger age (OR: 0.68, 95% CI: 0.547–0.844, P < 0.001), higher NLR (OR: 2.858, 95% CI: 2.295–3.559, P < 0.001), higher CEA (OR: 2.108, 95% CI: 1.671–2.661, P < 0.001), higher CA199 (OR: 1.844, 95% CI: 1.466–2.32, P < 0.001) and higher CA724 (OR: 2.348, 95% CI: 1.851–2.978, P < 0.001) were independent risk factors for GC metastasis (Table 4). In order to detect whether the prognostic factor is able in predicting metastatic evolution of GC, we conducted multivariate multiple logistic regression analyses. As shown in Table 5, unlike binary logistic regression that only considers whether GC has metastasized, we used TNM stage I as a reference in the multiple logistic regression analysis, and found that only CEA was an independent risk factor (higher CEA for reference, OR:0.424, 95% CI: 0.182–0.987, P = 0.047) in TNM stage II, that NLR (OR: 0.467, 95% CI: 0.266–0.818, P = 0.008), CEA (OR:0.342, 95% CI: 0.188–0.621, P < 0.001), CA19-9 (OR: 0.406, 95% CI: 0.234–0.703, P = 0.001) and CA72-4 (OR: 0.4, 95% CI: 0.216–0.741, P = 0.004) were independently associated with tumor progression in TNM stage III (high level of these parameters for reference), and that NLR (OR: 0.156, 95% CI: 0.092–0.265, P < 0.001), CEA (OR: 0.221, 95% CI: 0.124–0.394, P < 0.001), CA19-9 (OR: 0.28, 95% CI: 0.165–0.476, P < 0.001) and CA72-4 (OR: 0.228, 95% CI: 0.126–0.414, P < 0.001) showed significance in TNM stage IV (high level of these indicators for reference). Notably, age was not significant in the multiple logistic regression model.

**Table 4:** Univariate and multivariate binary logistic regression analyses of variables for GC metastasis.

	Univariate analysis			Multivariate analysis		
	Odds ratio	95% CI	P value	Odds ratio	95% CI	P value
**Age, years**						
< 60 vs. ≥ 60	0.739	0.609–0.898	0.002	0.68	0.547–0.844	< 0.001
**NLR**						
Low vs. high	3.375	2.748–4.145	< 0.001	2.858	2.295–3.559	< 0.001
**CEA**						
Low vs. high	3.026	2.455–3.729	< 0.001	2.108	1.671–2.661	< 0.001
**CA199**						
Low vs. high	2.813	2.289–3.455	< 0.001	1.844	1.466–2.32	< 0.001
**CA724**						
Low vs. high	3.385	2.722–4.211	< 0.001	2.348	1.851–2.978	< 0.001

The reference of age, NLR, CEA, CA19-9 and CA72-4 was age < 60, NLR < 2.91, CEA < 4.34, CA19-9 < 18.69 and CA72-4 < 6.96.

**Table 5:** Multivariate multiple logistic regression analyses of variables for GC metastasis.

	TNM stage II			TNM stage III			TNM stage IV		
	Odds ratio	95% CI	P value	Odds ratio	95% CI	P value	Odds ratio	95% CI	P value
NLR	0.765	0.32–1.827	0.547	0.467	0.266–0.818	0.008	0.156	0.092–0.265	< 0.001
CEA	0.424	0.182–0.987	0.047	0.342	0.188–0.621	< 0.001	0.221	0.124–0.394	< 0.001
CA19-9	0.87	0.364–2.08	0.755	0.406	0.234–0.703	0.001	0.28	0.165–0.476	< 0.001
CA72-4	1.238	0.42–3.65	0.699	0.4	0.216–0.741	0.004	0.228	0.126–0.414	< 0.001

For multiple logistic regression analyses the reference category is TNM stage I. The reference of NLR, CEA, CA19-9 and CA72-4 was NLR ≥ 2.91, CEA ≥ 4.34, CA19-9 ≥ 18.69 and CA72-4 ≥ 6.96.

*TNM* tumor-node-metastasis, *NLR* neutrophil–lymphocyte ratio, *CEA* carcinoembryonic antigen, *CA19-9* carbohydrate antigen 19-9, *CA72-4* carbohydrate antigen 72–4.

## Discussion

In our current study, we investigated the relationship between NLR and GC metastasis status. Our study illustrated that peripheral blood derived parameter NLR can more effectively predict GC metastases, including peritoneal, hepatic and osseous metastases, than CEA, CA19-9 and CA72-4. Non-invasiveness and high diagnostic efficiency makes NLR a novel GC marker to assist clinicians in evaluating and diagnosing GC metastasis.

Tumor-associated persistent inflammatory environment is induced by continuous cell renewal and proliferation^15^. Through crosstalk between malignant and non-malignant cells, the inflammatory tumor microenvironment ultimately promotes tumor progression and metastasis^4^. This driving force is derived from inflammatory-derived survival, growth, and pro-angiogenic factors and from extracellular matrix (ECM)-modifying enzymes that promote angiogenesis, invasion, and metastasis^16^. On the other hand, inflammation suppresses antitumor immune responses, which help tumors evade host immunosurveillance^17^. Tumor-associated neutrophils suppress T-cell’s function in a PD-L1-dependent fashion, and, in doing so, contribute to the GC progression in vitro and in vivo^18^. Changes in circulating neutrophils are also reflected in neutrophil infiltration of the tumor environment^19^. This enlightens us that the level of circulating neutrophils in tumor patients may reflect tumor metastasis status. In fact, circulating tumor-associated neutrophils in advanced cancer patients contributes to the survival of circulating tumor cell by suppressing peripheral leukocyte activation. With tumor progression, circulating neutrophils gradually increase and are converted into tumorigenic tumor-associated neutrophils, characterized by overexpression of CCL12, CXCL2 and Arg1 relative to naive neutrophils^20^. Neutrophils can also form a protective layer around circulating tumor cells to promote tumor metastasis^21^. These findings may have implications regarding the role of neutrophils in tumor metastasis prediction and tumor therapy.

Lymphocytes are part of cell-mediated immunity and play an important role in host anticancer defense mechanisms. Impaired lymphocyte function and reduced lymphocyte counts have been implicated in various cancers^22^. Perioperative lymphopenia is associated with poor prognosis in GC[^23,24^]. Mounting evidence demonstrated that both absolute lymphocytes counts (ALC) in peripheral blood and tumor-infiltrating lymphocytes (TIL) are associated with tumor progression. Low TIL is associated with poor prognosis in esophageal squamous cell carcinoma. Furthermore, ALC in peripheral blood is positively correlated with TIL^25^. However, in a study on breast cancer, ALC showed a negative correlation with TIL^26^. More research is needed to demonstrate the relationship between ALC and TIL.

Given that tumor patients are often accompanied by increased neutrophils and decreased lymphocytes, the predictive and prognostic role of NLR in tumors has been extensively studied. Most previous studies have either focused on high NLR as a useful predictor of long-term survival of cancer patients or explored the role of NLR in the assessment of lymph node metastasis[^11,14,27–30^]. Less attention has been paid to the predictive role of NLR in evaluating distant metastasis. NLR has been reported to be an effective predictor of distant metastasis after radical surgery for pancreatic ductal adenocarcinoma^21^. Nizhen Xu et al. demonstrated that elevated NLR is associated with a higher risk of metastasis in thyroid cancer patients^31^. Although the role of NLR in distant metastasis in GC patients has been reported, researchers did not compare the diagnostic efficacy of NLR with traditional tumor markers^32^. In our current study, we found that NLR was comparable or even superior to tumor markers in assessing distant metastases. The optimal cutoff value of NLR were determined to be 2.91 with a sensitivity of 48.5% and a specificity of 76.3%. In subgroup analysis (peritoneal metastases, osseous metastases and liver metastases), the diagnostic efficacy of combined NLR and GC markers was significantly higher than that of combined GC markers only. Both univariate and multivariate logistic regression analyses indicated that elevated NLR was independently associated with high risk of tumor metastasis. To further explore whether NLR increases gradually with tumor progression, we performed multiple logistic regression analysis based on TNM staging. Although only CEA was significant in TNM stage II, NLR, CEA, CA19-9 and CA72-4 were independent risk factors in TNM stage III and IV. This suggests that NLR can reflect the status of tumor progression in advanced GC. Cancer patients with elevated NLR, especially advanced patients, should be alert to tumor metastasis, especially the occurrence of micro-metastases. Further studies should be conducted to determine whether detection and targeting of neutrophils can improve GC prognosis and benefit clinical treatment.

In conclusion, our findings suggest that NLR is helpful in predicting the presence of distant metastases in GC patients. Therefore, physicians should pay more attention to patients with NLR > 2.91, and then conduct further examinations to detect metastases as early as possible.

## Material and methods

### Patients

We collected the clinical data in The First Affiliated Hospital of Xi’an Jiaotong University between December 2010 and December 2018. Patients were excluded if they met one of the following criteria: (1) concurrent or secondary to other malignancies; (2) Infection; (3) Hematological disease or blood transfusion within the past 3 months; (4) No pre-treatment laboratory data. All patients had pretreatment hematological values and conventional GC biomarkers. At last, 1,667 patients first diagnosed with GC were enrolled in our study. Data evaluated included age at diagnosis, preoperative complete blood counts, Carcinoembryonic antigen (CEA), Carbohydrate antigen 19-9 (CA19-9) and Carbohydrate antigen 72-4 (CA72-4). Blood parameters were those closest to the time of treatment. Informed consent was obtained from all subjects and/or their legal guardian(s). All methods were performed in accordance with the relevant guidelines and regulations. This study was approved by the Ethics Committee of First Affiliated Hospital of Xi'an Jiaotong University.

### Statistical analysis

We divided patients into metastatic (N = 743) and non-metastatic (N = 924) groups according to whether GC has metastasized or not. Counting variables are expressed as frequency (percentage) and compared using the Chi-square test or Fisher exact test. Non-normal continuous variables are expressed as the median [interquartile range (IQR)] and compared with log-rank tests, while continuous normal variates were presented as mean ± standard deviation and compared using Student's t-tests. The optimal cut-off values for NLR and GC biomarkers were obtained by ROC analyses. The predicted probability of the joint ROC curve analysis is obtained by binary logistic regression. Area under ROC (AUC) were compared using DeLong's test. We analyzed the correlation of NLR and other clinical indicators by Spearman’s correlation. Multiple logistic regression was performed to assess the relationship between explanatory variables and GC metastasis.

Statistical analysis and plotting were performed with IBM SPSS Statistics version 20.0 RRID:SCR_016479. DeLong's test was performed using MedCalc version 19.4.1 RRID:SCR_015044. 2-sided P < 0.05 were considered statistical significantly.

## Supplementary Information

Supplementary Tables.

## Data Availability

The data generated in this study are available upon request from the corresponding author.
