# Supplementary material for: Predictive value of neutrophil-to-lymphocyte ratio for distant metastasis in gastric cancer patients
Source: Sci Rep. 2022 Jun 17;12:10269. doi: 10.1038/s41598-022-14379-4 (PMC9205918; doi:10.1038/s41598-022-14379-4)
Supplement: Supplementary file 1 — Supplementary Tables. [file 41598_2022_14379_MOESM1_ESM.pdf]

Predictive value of neutrophil-to-lymphocyte ratio for distant metastasis in gastric cancer patients

Xin Zhang[1M.D.], Xuan Wang[1 M.D.], Wenxing Li[1M.D.], Tuanhe Sun[1M.D.], Dongmei Diao[#1 M.D., Ph.D.] and Chengxue Dang[#1M.D., Ph.D.],
[1]Department of Oncology Surgery, First Affiliated Hospital of Xi'an Jiaotong University, Xi'an, China;
[#] Corresponding Author: Dr. Dongmei Diao and Prof. Chengxue Dang, Ph.D., M.D., Department of Surgical Oncology, First Affiliated Hospital Medical college Xi'an Jiaotong University, 277 West Yanta Road, Xi'an, Shaanxi 710061, P.R. China. Tel: 86-29-85324612, Fax: 86-29-85324612, E-mail: diaomei310@mail.xjtu.edu.cn; **dangchengxue@mail.xjtu.edu.cn.**
[#] these authors contributed equally to this work.

Supplementary Table 1 AUC and cut points of diagnostic indicators at the maximum of Youden index for peritoneal metastasis (N=163)

	AUC	95% CI	Cut-off	Sen	Spe	Youden index	PPV	NPV	P value
NLR	0.704	0.662-0.747	2.69	0.648	0.684	0.332	0.648	0.683	0.003
CEA	0.557	0.507-0.608	5.09	0.352	0.8	0.152	0.346	0.8	<0.001
CA199	0.626	0.573-0.679	48.84	0.377	0.88	0.257	0.377	0.88	<0.001
CA724	0.694	0.648-0.739	3.41	0.654	0.655	0.309	0.654	0.655	0.013
P	0.757	0.715-0.798	0.125	0.667	0.773	0.44	0.66	0.773	Ref

AUC, area under receiver operating characteristics; CI, confidence interval; Sen, sensitivity; Spe, specificity; PPV, positive predictive value; NPV, negative predictive value; NLR, neutrophil-to-lymphocyte ratio; P, P value for comparison of AUC of reference with other indicators; P, Prediction probability that was obtained by binary logistic regression of CEA, CA199, CA724 and NLR.

Supplementary Table 2 AUC and the cut points of diagnostic indicators at the maximum of Youden index for osseous metastasis (N=119)

	AUC	95% CI	Cut-off	Sen	Spe	Youden index	PPV	NPV	P value
NLR	0.653	0.599-0.707	2.36	0.639	0.601	0.24	0.661	0.600	0.313
CEA	0.65	0.594-0.707	3.82	0.538	0.727	0.265	0.563	0.727	0.514
CA199	0.555	0.498-0.612	67.43	0.218	0.906	0.124	0.243	0.906	<0.001
CA724	0.586	0.524-0.648	2.75	0.597	0.589	0.186	0.603	0.589	0.017
P	0.671	0.614-0.727	0.1	0.521	0.774	0.295	0.529	0.763	Ref

P, P value for comparison of AUC of reference with other indicators; P, Prediction probability that was obtained by binary logistic regression of CEA, CA199, CA724 and NLR.

Supplementary Table 3 AUC and the cut points of diagnostic indicators at the maximum of Youden index for hepatic metastasis (N=192)

	AUC	95% CI	Cut-off	Sen	Spe	Youden index	PPV	NPV	P value
NLR	0.676	0.633-0.718	2.71	0.62	0.687	0.307	0.600	0.687	<0.001
CEA	0.733	0.693-0.774	4.34	0.615	0.764	0.379	0.651	0.764	0.041
CA199	0.713	0.669-0.757	18.69	0.615	0.742	0.357	0.614	0.738	0.002
CA724	0.626	0.58-0.673	7.76	0.396	0.828	0.224	0.395	0.828	<0.001
P	0.777	0.74-0.815	0.133	0.74	0.699	0.439	0.740	0.688	Ref

P, P value for comparison of AUC of reference with other indicators; P, Prediction probability that was obtained by binary logistic regression of CEA, CA199, CA724 and NLR.

Supplementary Table 4 AUC and the cut points of diagnostic indicators at the maximum of Youden index for multisite metastases (N=122)

	AUC	95% CI	Cut-off	Sen	Spe	Youden index	PPV	NPV	P value
NLR	0.718	0.67-0.767	2.64	0.664	0.676	0.34	0.213	0.938	0.005
CEA	0.629	0.572-0.685	4.365	0.459	0.766	0.225	0.206	0.915	<0.001
CA199	0.677	0.622-0.732	19.44	0.549	0.746	0.295	0.223	0.926	0.002
CA724	0.709	0.657-0.761	5.39	0.59	0.745	0.335	0.235	0.932	0.066
P1	0.742	0.693-0.79	0.095	0.648	0.735	0.383	0.244	0.94	0.316
P2	0.765	0.719-0.811	0.097	0.68	0.781	0.461	0.291	0.949	Ref

P, P value for comparison of AUC of reference with other indicators; P1, Prediction probability was obtained by binary logistic regression of CEA, CA199 and CA724. P2, Prediction probability that was obtained by binary logistic regression of CEA, CA199, CA724 and NLR.
